# Bayesian networks and imaging-derived phenotypes highlight the role of fat deposition in COVID-19 hospitalisation risk

**DOI:** 10.3389/fbinf.2023.1163430

**Published:** 2023-05-24

**Authors:** T. Waddell, A. I. L. Namburete, P. Duckworth, N. Eichert, H. Thomaides-Brears, D. J. Cuthbertson, J. P. Despres, M. Brady

**Affiliations:** ^1^ Department of Engineering Science, The University of Oxford, Oxford, United Kingdom; ^2^ Perspectum Ltd., Oxford, United Kingdom; ^3^ Department of Computer Science, University of Oxford, Oxford, United Kingdom; ^4^ Oxford Robotics Institute, The University of Oxford, Oxford, United Kingdom; ^5^ Department of Cardiovascular and Metabolic Medicine, Institute of Life Course and Medical Sciences, University of Liverpool, Liverpool, United Kingdom; ^6^ Liverpool University Hospitals NHS Foundation Trust, Liverpool, United Kingdom; ^7^ Scientific director of VITAM – Research Center for Sustainable Health, Laval University, Quebec, QC, Canada

**Keywords:** Bayesian networks, probabilistic reasoning, ectopic fat, COVID-19, hospitalisation

## Abstract

**Objective:** Obesity is a significant risk factor for adverse outcomes following coronavirus infection (COVID-19). However, BMI fails to capture differences in the body fat distribution, the critical driver of metabolic health. Conventional statistical methodologies lack functionality to investigate the *causality* between fat distribution and disease outcomes.

**Methods:** We applied Bayesian network (BN) modelling to explore the mechanistic link between body fat deposition and hospitalisation risk in 459 participants with COVID-19 (395 non-hospitalised and 64 hospitalised). MRI-derived measures of visceral adipose tissue (VAT), subcutaneous adipose tissue (SAT), and liver fat were included. Conditional probability queries were performed to estimate the probability of hospitalisation after fixing the value of specific network variables.

**Results:** The probability of hospitalisation was 18% higher in people living with obesity than those with normal weight, with elevated VAT being the primary determinant of obesity-related risk. Across all BMI categories, elevated VAT and liver fat (>10%) were associated with a 39% mean increase in the probability of hospitalisation. Among those with normal weight, reducing liver fat content from >10% to <5% reduced hospitalisation risk by 29%.

**Conclusion:** Body fat distribution is a critical determinant of COVID-19 hospitalisation risk. BN modelling and probabilistic inferences assist our understanding of the mechanistic associations between imaging-derived phenotypes and COVID-19 hospitalisation risk.

## Introduction

Obesity is currently one of the leading global causes of poor health, with 28% and 41% of adults in the United Kingdom and US, respectively, being classified as living with obesity (Powell-Wiley et al., 2021; NHS Digital, 2019). Global obesity prevalence has increased by 5% since 2010, and by 2030 more than one billion people will be living with obesity (Lobstein et al., 2022). The obesity healthcare crisis has been further exacerbated with the spread of the SARS-CoV-2 virus, the coronavirus pandemic (COVID-19), and the interaction between obesity and COVID-19. Specifically, obesity has been repeatedly highlighted as a significant risk factor for adverse outcomes following COVID-19, including the need for mechanical ventilation, hospitalisation, and mortality (Sattar et al., 2020; Soeroto et al., 2020; Freuer et al., 2021; Sawadogo et al., 2022).

Additionally, people living with obesity are known to be at a significantly greater risk of type-2 diabetes (T2D), cardiovascular disease, and non-alcoholic fatty liver disease (NAFLD) (Holman et al., 2011), each of which is associated with worse COVID-19 outcomes, particularly when these conditions are co-prevalent (O’hearn et al., 2021; Ando et al., 2021; Hebbard et al., 2021; Roca-Fernández et al., 2021).

Although widely used as an objective measure of obesity, BMI poorly reflects the body fat distribution; indeed, some have suggested that the waist circumference may better reflect the body composition (Ross et al., 2020). The BMI confounds all body components (e.g., visceral fat and skeletal muscle) into a single measure (weight/height^2^). Independent of BMI, an elevated visceral adipose tissue (VAT)/subcutaneous adipose tissue (SAT) ratio is a significant predictor of poorer COVID-19 prognosis (Bunnell et al., 2021; Ogata et al., 2021), and people with “metabolically healthy obesity” have a lower risk of T2D than those with “metabolically unhealthy obesity” (Hinnouho et al., 2015). Furthermore, in BMI-matched people, those with T2D have demonstrated significantly elevated VAT and liver fat deposition (Waddell et al., 2022; Levelt et al., 2016). Ectopic fat deposition is a critical factor in metabolic health and adverse COVID-19 outcomes. Fundamentally, the biological heterogeneity of obesity sub-phenotypes is not sufficiently captured by the BMI alone.

Bayesian networks (BNs) are a powerful tool for visualising complex systems, modelling uncertainty, and, under certain assumptions, specifying causal relationships. BNs, combined with probabilistic reasoning, can be used to estimate the probability of an event (for example, hospitalisation caused by COVID) after fixing the values of specific network variables (e.g., obesity, visceral, or liver fat (high *vs.* low for each)) and performing conditional probability queries. For example, Li et al. (2020) estimated acute kidney injury risk in patients with concurrent gastrointestinal cancers, while Xie et al. (2017) and Fuster-Parra et al. (2016) predicted the onset of T2D and cardiovascular disease, respectively. While BNs have been applied to model the COVID-19 risk with great success (Butcher and Fenton, 2020; Neil et al., 2020; Fenton et al., 2021), they are yet to be applied for studying the body fat distribution and risk of COVID-19 hospitalisation.

We describe how MRI-derived phenotypes assess volumes of different fat deposits, including liver fat and body composition, combined with BN modelling to capture *conditional* dependencies, and enable the phenotypic characterisation of the highest risk of metabolic phenotypes to be hospitalised following COVID-19. We also compare the predictive performance of BNs against traditional classification algorithms.

## Materials and methods

### Data collection and preparation

Anthropometric, demographic, and imaging data were extracted from the COVERSCAN study (NCT04369807) that recruited participants with confirmed COVID-19 between April 2020 and October 2021. All participants underwent an abdominal MRI assessment that included liver and body composition. In total, data from 466 participants with the necessary imaging data available at the time of analysis were collected; after removing missing data entries, 459 were selected for further analysis (395 non-hospitalised and 64 hospitalised). See Supplementary Figure S5 for a full cohort flow diagram containing further details.

### Bayesian network construction

BNs are a class of graphical models that encode probabilistic relationships in the form of a directed acyclic graph (DAG). Formally, a DAG is expressed as G = (V, E), where V = {X_1_, X_2_, …, X_n_} denotes the random variables of interest (in the present case, participant biomarkers) and E is a set of directed edges relating pairs of variables in V. The directionality of an edge from $X_{i}$ to $X_{j}$ captures the flow of *information* between those two variables, where the value of $X_{j}$ is conditionally dependent on the value of $X_{i}$ . Concretely, the structure of a DAG is comprised of three types of connections between variables: chains, forks, and colliders. Together, these allow the reader to conveniently (and visually) detect interdependencies within the data. See Supplementary Section S1 for explanations of BNs and probabilistic inference.

The following variables were selected for constructing the BN: smoking status (never smoked, current smoker, and past smoker), hospitalisation status (1 [hospitalised]/0 [non-hospitalised]), liver fat (proton density fat fraction [PDFF] %), visceral adipose tissue (cm^2^), subcutaneous adipose tissue (SAT) (cm^2^), skeletal muscle (cm^2^), gender (male/female), BMI (kg/m^2^), and age (yrs). Body composition was examined from a 2D MR slice positioned at the third lumbar (L3) vertebra, and VAT, SAT, and skeletal muscle were measured based on manual delineations by trained analysts, see Supplementary Figure S4. The measures of skeletal muscles were then indexed to the participant’s height to produce a measure of the skeletal muscle index (SMI) (cm^2^/m^2^). All continuous variables were discretised based on pre-defined clinical thresholds; for example, the BMI was discretised into obesity categories: normal weight (BMI <25 kg/m^2^), overweight (BMI 25–30 kg/m^2^), and obese (BMI >30 kg/m^2^). See Supplementary Table S1 for a full overview of discretisation thresholds.

**FIGURE 1:**
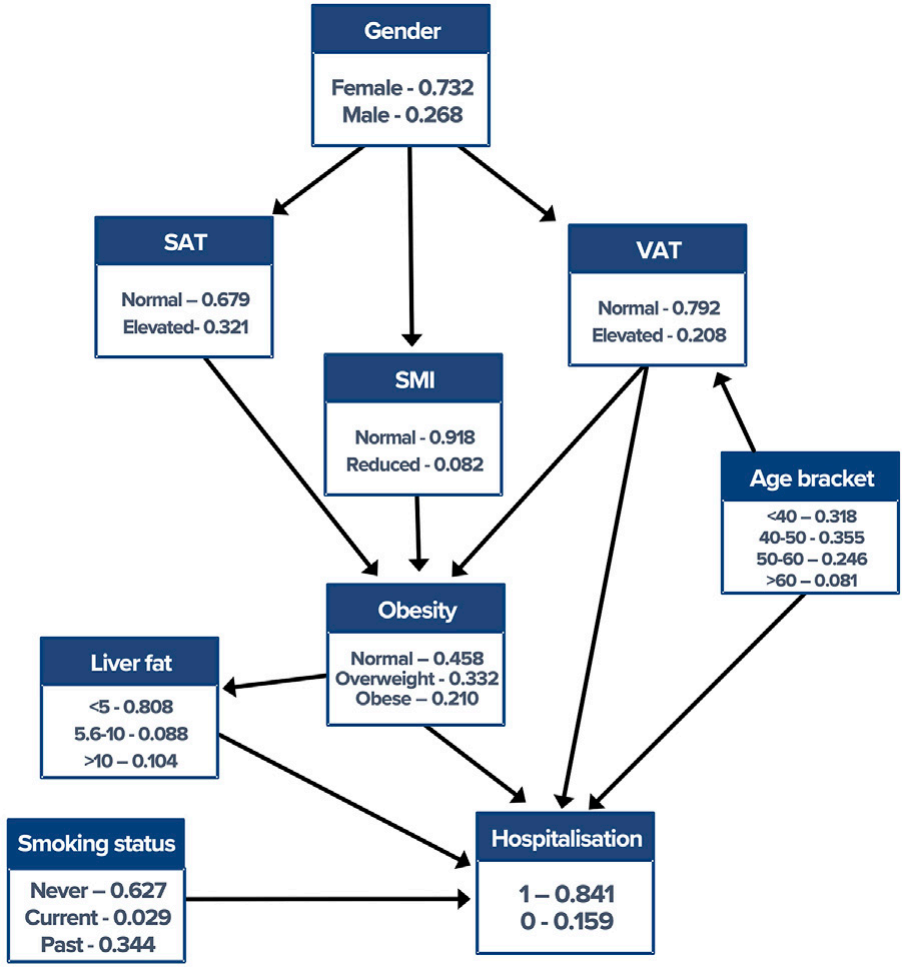
Semantic Bayesian network with corresponding conditional probability tables. Edge directionality makes the direction of conditional dependence explicit. VAT, visceral adipose tissue; SMI, skeletal muscle index; SAT, subcutaneous adipose tissue.

BN construction and inference were completed using the “bnlearn” package (Scutari, 2010) and visualised using “graphviz” within the R software platform (version 3.6.1). The score-based hill-climbing structure learning algorithm with the Bayesian information criterion (BIC) provided the initial network construction. The network was then adjusted by removing or reversing nonsensical edges and inserting edges based on domain knowledge in collaboration with medical experts, rendering what is referred to as a “semantic” network. Figure 1 shows the final network. Crucially, the incorporation of clinical knowledge in the network enables the modelling of *causality* between variables, for the presence and direction of edges being not simply bias dependencies within the dataset.

### Probabilistic inference

Probabilistic inference allows the user to pose counterfactual “what if” questions by intervening in the network. This is performed by “fixing” the value(s) of specific variables (evidence) and then estimating the probability of an “event,” given the evidence. Specifically, in our work, this included estimating the probability of hospitalisation, given an “evidence list” containing the fixed values of network variables such as liver fat or VAT. For example, the probability of hospitalisation was estimated after fixing the values of “liver fat” to normal liver fat (<5%), mild steatosis (5%–10%), and severe steatosis (>10%), allowing the direct effect of elevated liver fat on hospitalisation risk to be inferred. Conditional probability queries were performed using the “cpquery” function in bnlearn and estimated using likelihood weighting algorithm, a Monte Carlo approximation technique that uses importance sampling from the “mutilated network.” This algorithm was selected due to the relatively low sample size and confirmed hospitalisations.

### Prediction of the hospitalisation status

The following statistical and machine learning (ML) classification algorithms were used as a comparison for predicting the hospitalisation status: logistic regression, Naïve Bayes, and decision tree. These were implemented within R using “brglm2,” “klaR,” and “tree” packages, respectively. The dataset was split into train (internal) [*n* = 46 (six hospitalised and 40 non-hospitalised)] and test (validation) (*n* = 413 [58 hospitalised and 355 non-hospitalised]) validation cohorts, adopting a 10:90 split while retaining equal proportions of hospitalised participants in each cohort. Given the relatively low number of hospitalisations, this data split was selected to avoid overfitting the prediction models and to minimise variance in prediction results. The area under the receiver operating characteristic curves (AUC) was reported to measure model performance, see Supplementary Table S2 for additional performance measures.

### Statistical analyses

All statistical analyses used the R software platform (version 3.6.1). Descriptive statistics, showing median [inter-quartile range], were reported to summarise population characteristics. Adopting a significance threshold at *p* < 0.05, Wilcoxon and X^2^ tests revealed that hospitalised participants were significantly older (*p* < 0.001) and had significantly elevated measures of BMI (*p* = 0.042), liver fat (*p* = 0.0028), and VAT (*p* < 0.001). See Table 1.

**TABLE 1:** Population characteristics between hospitalised and non-hospitalised participants. Data are represented as median [IQR] and significant *p*-values in bold.

Participant variable	Hospitalised (*n* = 64)	Non-hospitalised (*n* = 395)	*p*-value
Age (yrs)	50 [43–57]	43 [37–51]	**0.00035**
Gender (% M)	35%	33%	0.8
BMI (kg/m^2^)	27 [23–32]	25 [22–28]	**0.042**
Liver fat (% PDFF)	3 [2.2–6.7]	2.4 [1.9–3.9]	**0.0028**
VAT (cm^2^)	120 [66–203]	85 [47–131]	**0.00077**
SAT (cm^2^)	237 [141–315]	205 [136–305]	0.19
SMI (cm^2^/m^2^)	45 [39–49]	43 [38–49]	0.31

bold values denotes significance (p <0.05).

## Results

### Model prediction

**FIGURE 2:**
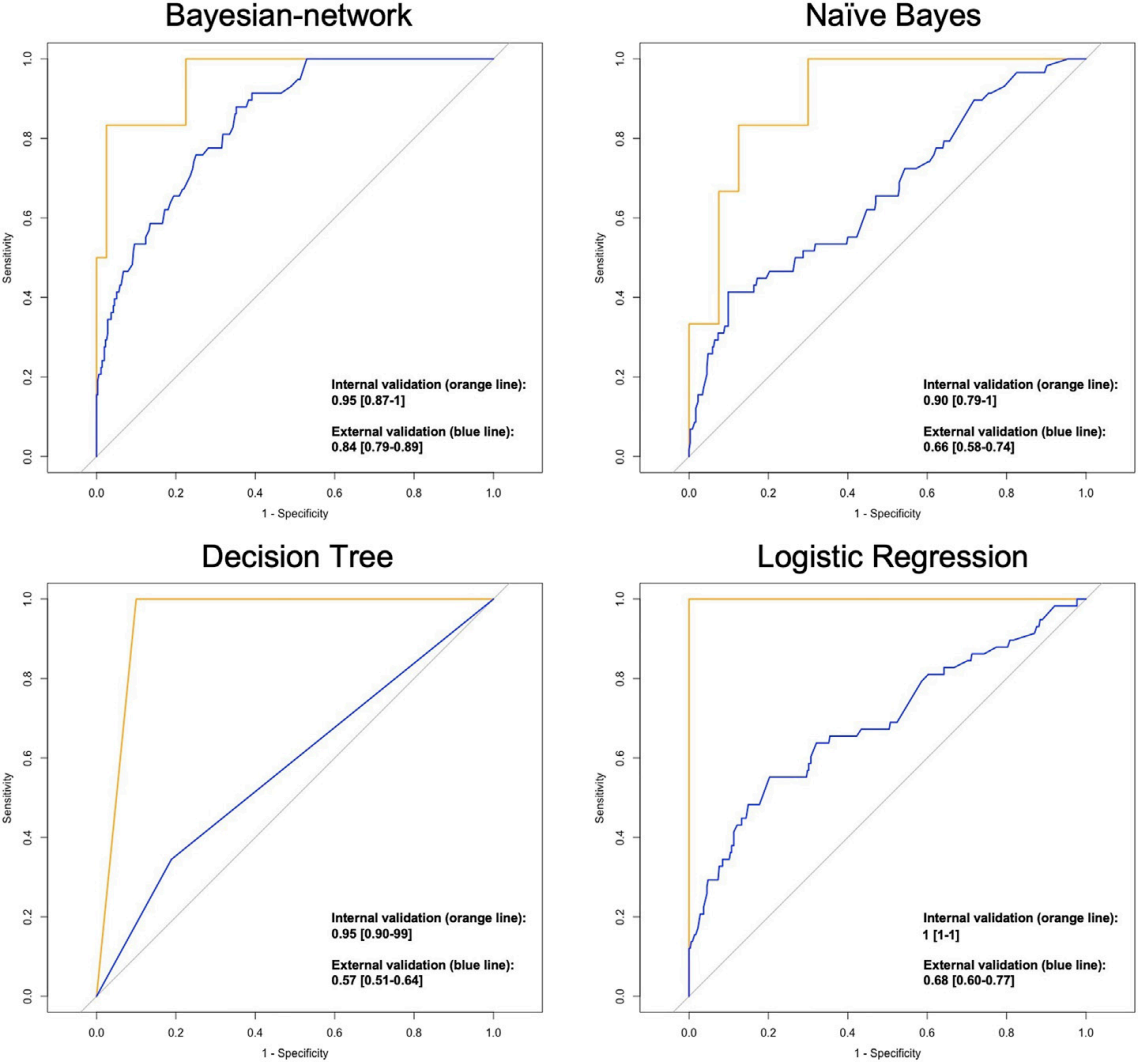
ROC plots indicating the performance of classification models in predicting the hospitalisation status AUC [95% CI].

ROC curves indicating model performance are shown in Figure 2. Overall, the performance of the validation cohort was the highest within the BN model [AUC (95% CI)] [0.84 (0.79–0.89)], followed by logistic regression [0.68 (0.60–0.77)], Naïve Bayes [0.66 (0.58–0.74)], and decision tree [0.57 (0.51–0.64)].

### Estimation of hospitalisation using probabilistic reasoning

The baseline probability of hospitalisation caused by acute COVID-19 was 15%. “Intervening” on a variable within the BN means that we assign it a specific value. For example, the variable “Obesity” can be assigned the values normal weight, overweight, or obese. The effect of such an intervention is then propagated throughout the network, where the variables conditionally dependent on the intervened variable(s) are updated to reflect the specified evidence.

We first intervened on the variable “Obesity” only, observing an 11%, 13%, and 29% probability of hospitalisation when the value was set, respectively, to normal weight, overweight, and obese. This equates to an 18% greater probability of hospitalisation when “Obesity” was set to obese than normal weight.

To ascertain what component of the body composition was driving the elevated obesity risk, we examined the probability of living with obesity given varying measures of body composition. We observed that elevated VAT doubled the probability of living with obesity compared to elevated SAT (50% *vs.* 24%), identifying elevated VAT as the primary determinant of obesity. We then intervened on the variables “Liver fat” and “VAT” without fixing the value of “Obesity.” Elevated VAT resulted in a 31% probability of hospitalisation; this was reduced by 20% when specifying non-elevated VAT, which elicited an 11% probability of hospitalisation. We observed a “Liver fat” value of <5% (normal), 5%–10% (mild steatosis), and >10% (severe steatosis) that resulted in a 12%, 25%, and 30% probability of hospitalisation, respectively.

We then examined the influence of VAT and liver fat on hospitalisation risk across each BMI-based category. We first estimated the probability of hospitalisation with normal measures of liver fat (<5%) and VAT, here referred to as our “baseline” measurement. Next, the value of the variables “VAT” and “Liver fat” was individually fixed to ascertain the direct effect of each variable state on hospitalisation risk, see Table 2.

**TABLE 2:** Probability of hospitalisation (%) and probability change (+/− %) across obesity categories. The probability of hospitalisation was estimated, given the fixed values of the network variables as set in the column headings.

	Liver fat <5 (%) normal VAT (baseline)	Liver fat 5%–10%	Liver fat >10%	Elevated VAT	Elevated VAT and liver fat >10%
Normal weight (*n* = 210)	10	26% (+16%)	39% (+29%)	27% (+17%)	45% (+35%)
Overweight (*n* = 148)	8	2% (−6%)	9% (+1%)	31% (+23%)	41% (+33%)
Obesity (*n* = 101)	14	55% (+41%)	35 (+21%)	25% (+11%)	31% (+17%)

## Discussion

Obesity is a significant risk factor of hospitalisation following COVID-19; however, the use of BMI as a measure of body fat distribution is intrinsically limited. We show how applying BN modelling and probabilistic reasoning to MRI-derived measures of liver fat and body composition enables the associations between obesity and hospitalisation risk to be unravelled.

We first demonstrate an 18% greater probability of hospitalisation in people with obesity *vs.* people with normal weight, with elevated VAT being the primary determinant of obesity. We postulate that the association between obesity and hospitalisation following COVID-19 is primarily driven by VAT deposition rather than SAT. This is consistent with (14) who reported a higher VAT/SAT ratio being significantly predictive of adverse outcomes following COVID-19, independent of the BMI status.

Except for the case when “Obesity” was set to overweight, we demonstrate that liver fat measures of 5%–10% and >10% consistently increased the probability of hospitalisation by an average of 29% and 25%, respectively. Similar work on data from the UK Biobank by (12) reported a fivefold greater risk of hospitalisation in people with obesity with concurrent NAFLD *vs.* those with obesity but without NAFLD. Furthermore, across all obesity categories, both elevated VAT and liver fat together caused an average increase in the probability of hospitalisation of 39%. Taken together, these results emphasise the role of ectopic fat deposition in driving hospitalisation risk, which is not captured through the use of the BMI alone. Postulated mechanisms between elevated VAT and poorer COVID-19 prognosis include an overexpression of proinflammatory cytokines and increased lipolysis, leading to epithelial injury (Cartin-Ceba et al., 2022; Colleluori et al., 2022).

Probabilistic inference can be used to estimate patient outcomes based on changes in clinical variables following treatment. For example, we show that even under normal weight settings, reducing liver fat content from >10% to <5% reduced the probability of hospitalisation by 29%. Therefore, in this population, therapies known to elicit favourable changes in liver health without requiring significant changes in body weight, such as the Mediterranean diet or exercise training, should be considered a viable therapeutic option (Chakravarthy et al., 2020). A >10% to <5% reduction in liver fat has been demonstrated in people with obesity following bariatric surgery (Luo et al., 2018), while treatment with GLP-1 receptor agonists has shown to reduce liver fat, VAT, and associated metabolic markers such as hyperglycaemia (Flint et al., 2021; Gadde and Heymsfield, 2021). Most significantly, while not directly explored here, our work highlights BNs as promising tools for modelling and estimating a variety of different treatment outcomes, allowing personalised treatment strategies and expectations to be formulated.

We acknowledge the limitations of the present analysis. First, MRI-derived measures of body composition were derived from a 2D slice positioned at the third lumbar vertebra, which although being an established technique for measuring body composition (Zaffina et al., 2022), leaves total body fat distribution open to generalisation. Second, hospitalisation risk following COVID-19 is notably complex and involves many factors, such as biochemical pathways, that were not investigated here. Future works will seek to incorporate biochemical pathways and circulating biomarkers into the network, providing a more comprehensive assessment of metabolic health and hospitalisation risk.

In conclusion, we applied BN modelling and probabilistic inference to study the association between body composition, liver fat deposition, and hospitalisation risk following COVID-19. We show that elevated VAT and liver fat both increase the probability of hospitalisation and illustrate how BNs can be applied to estimate counterfactual patient outcomes in the context of biological systems. Future works could incorporate treatment outcome data into the Bayesian network to predict optimal therapeutic options for patients to reduce the risk of hospitalisation caused by acute COVID-19.

## Data Availability

The raw data supporting the conclusion of this article will be made available by the authors, without undue reservation.

## References

[B1] Ando W. Horii T. Uematsu T. Hanaki H. Atsuda K. Otori K. (2021). Impact of overlapping risks of type 2 diabetes and obesity on coronavirus disease severity in the United States. Sci. Rep. 11 (1), 17968–8. 10.1038/s41598-021-96720-x 34504112 PMC8429758

[B2] Bunnell K. M. Thaweethai T. Buckless C. Shinnick D. J. Torriani M. Foulkes A. S. (2021). Body composition predictors of outcome in patients with COVID-19. Int. J. Obes. 45 (10), 2238–2243. 10.1038/s41366-021-00907-1 PMC8267764 34244597

[B3] Butcher R. Fenton N. (2020). Extending the range of symptoms in a bayesian network for the predictive diagnosis of COVID-19. medRxiv, 2020. 10.1101/2020.10.22.20217554

[B4] Cartin-Ceba R. Khatua B. El-Kurdi B. Trivedi S. Kostenko S. Imam Z. (2022). Evidence showing lipotoxicity worsens outcomes in Covid-19 patients and insights about the underlying mechanisms. Iscience 25 (5), 104322. 10.1016/j.isci.2022.104322 35502320 PMC9045865

[B5] Chakravarthy M. V. Waddell T. Banerjee R. Guess N. (2020). Nutrition and nonalcoholic fatty liver disease: Current perspectives. Gastroenterol. Clin. 49 (1), 63–94. 10.1016/j.gtc.2019.09.003 32033765

[B6] Colleluori G. Graciotti L. Pesaresi M. Di Vincenzo A. Perugini J. Di Mercurio E. (2022). Visceral fat inflammation and fat embolism are associated with lung’s lipidic hyaline membranes in subjects with COVID-19. Int. J. Obes. 46 (5), 1009–1017. 10.1038/s41366-022-01071-w PMC8790008 35082385

[B7] Fenton N. E. McLachlan S. Lucas P. Dube K. Hitman G. A. Osman M. (2021). A Bayesian network model for personalised COVID19 risk assessment and contact tracing. MedRxiv, 2020–2107.

[B8] Flint A. Andersen G. Hockings P. Johansson L. Morsing A. Sundby Palle M. (2021). Randomised clinical trial: Semaglutide versus placebo reduced liver steatosis but not liver stiffness in subjects with non‐alcoholic fatty liver disease assessed by magnetic resonance imaging. Alimentary Pharmacol. Ther. 54 (9), 1150–1161. 10.1111/apt.16608 PMC9292692 34570916

[B9] Freuer D. Linseisen J. Meisinger C. (2021). Impact of body composition on COVID-19 susceptibility and severity: A two-sample multivariable mendelian randomization study. Metabolism 118, 154732. 10.1016/j.metabol.2021.154732 33631142 PMC7900753

[B10] Fuster-Parra P. Tauler P. Bennasar-Veny M. Ligęza A. Lopez-Gonzalez A. A. Aguiló A. (2016). Bayesian network modeling: A case study of an epidemiologic system analysis of cardiovascular risk. Comput. methods programs Biomed. 126, 128–142. 10.1016/j.cmpb.2015.12.010 26777431

[B11] Gadde K. M. Heymsfield S. B. (2021). Targeting visceral adiposity with pharmacotherapy. lancet 9, 551–552. 10.1016/s2213-8587(21)00204-7 34358470

[B12] Hebbard C. Lee B. Katare R. Garikipati V. N. S. (2021). Diabetes, heart failure, and COVID-19: An update. Front. Physiology 12, 706185. 10.3389/fphys.2021.706185 PMC8554151 34721055

[B13] Hinnouho G.-M. Czernichow S. Dugravot A. Nabi H. Brunner E. J. Kivimaki M. (2015). Metabolically healthy obesity and the risk of cardiovascular disease and type 2 diabetes: The whitehall ii cohort study. Eur. heart J. 36 (9), 551–559. 10.1093/eurheartj/ehu123 24670711 PMC4344958

[B14] Holman N. Forouhi N. G. Goyder E. Wild S. H. (2011). The association of public health observatories (APHO) diabetes prevalence model: Estimates of total diabetes prevalence for england, 2010–2030. Diabet. Med. 28 (5), 575–582. 10.1111/j.1464-5491.2010.03216.x 21480968

[B15] Levelt E. Pavlides M. Banerjee R. Mahmod M. Kelly C. Sellwood J. (2016). Ectopic and visceral fat deposition in lean and obese patients with type 2 diabetes. J. Am. Coll. Cardiol. 68 (1), 53–63. 10.1016/j.jacc.2016.03.597 27364051 PMC4925621

[B16] Li Y. Chen X. Shen Z. Wang Y. Hu J. Zhang Y. (2020). Prediction models for acute kidney injury in patients with gastrointestinal cancers: A real-world study based on bayesian networks. Ren. Fail. 42 (1), 869–876. 10.1080/0886022x.2020.1810068 32838613 PMC7472473

[B17] Lobstein T. Brinsden H. Neveux M. (2022). World obesity atlas 2022. United Kingdom: World Obesity Federation. Available at: https://policycommons.net/artifacts/2266990/world_obesity_atlas_2022_web/3026660/.

[B18] Luo R. B. Suzuki T. Hooker J. C. Covarrubias Y. Schlein A. Liu S. (2018). How bariatric surgery affects liver volume and fat density in NAFLD patients. Surg. Endosc. 32 (4), 1675–1682. 10.1007/s00464-017-5846-9 29218660 PMC6690434

[B19] Neil M. Fenton N. Osman M. McLachlan S. (2020). Bayesian network analysis of Covid-19 data reveals higher infection prevalence rates and lower fatality rates than widely reported. J. Risk Res. 23 (7-8), 866–879. 10.1080/13669877.2020.1778771

[B20] NHS Digital (2019). Health survey for england. Available at: https://www.gov.uk/government/statistics/health-survey-for-england-2018 (Accessed June 1, 2022).

[B21] Ogata H. Mori M. Jingushi Y. Matsuzaki H. Katahira K. Ishimatsu A. (2021). Impact of visceral fat on the prognosis of coronavirus disease 2019: An observational cohort study. BMC Infect. Dis. 21 (1), 1240–1248. 10.1186/s12879-021-06958-z 34893021 PMC8660963

[B22] O’hearn M. Liu J. Cudhea F. Micha R. Mozaffarian D. (2021). Coronavirus disease 2019 hospitalizations attributable to cardiometabolic conditions in the United States: A comparative risk assessment analysis. J. Am. Heart Assoc. 10 (5), e019259. 10.1161/jaha.120.019259 33629868 PMC8174244

[B23] Powell-Wiley T. M. Poirier P. Burke L. E. Després J. P. Gordon-Larsen P. Lavie C. J. (2021). Obesity and cardiovascular disease: A scientific statement from the American heart association. Circulation 143 (21), e984–e1010. 10.1161/cir.0000000000000973 33882682 PMC8493650

[B24] Roca-Fernández A. Dennis A. Nicholls R. McGonigle J. Kelly M. Banerjee R. (2021). Hepatic steatosis, rather than underlying obesity, increases the risk of infection and hospitalization for COVID-19. Front. Med. 8, 636637. 10.3389/fmed.2021.636637 PMC8039134 33855033

[B25] Ross R. Neeland I. J. Yamashita S. Shai I. Seidell J. Magni P. (2020). Waist circumference as a vital sign in clinical practice: A consensus statement from the IAS and ICCR working Group on visceral obesity. Nat. Rev. Endocrinol. 16 (3), 177–189. 10.1038/s41574-019-0310-7 32020062 PMC7027970

[B26] Sattar N. Ho F. K. Gill J. M. Ghouri N. Gray S. R. Celis-Morales C. A. (2020). BMI and future risk for COVID-19 infection and death across sex, age and ethnicity: Preliminary findings from UK biobank. Diabetes & Metabolic Syndrome Clin. Res. Rev. 14 (5), 1149–1151. 10.1016/j.dsx.2020.06.060 PMC7326434 32668401

[B27] Sawadogo W. Tsegaye M. Gizaw A. Adera T. (2022). Overweight and obesity as risk factors for COVID-19-associated hospitalisations and death: Systematic review and meta-analysis. BMJ Nutr. Prev. Health 5, 10–18. 10.1136/bmjnph-2021-000375 PMC8783972 35814718

[B28] Scutari M. (2010). Learning bayesian networks with the bnlearn R package. J. Stat. Softw. 35 (3), 1–22. 10.18637/jss.v035.i03 21603108

[B29] Soeroto A. Y. Soetedjo N. N. Purwiga A. Santoso P. Kulsum I. D. Suryadinata H. (2020). Effect of increased BMI and obesity on the outcome of COVID-19 adult patients: A systematic review and meta-analysis. Diabetes & Metabolic Syndrome Clin. Res. Rev. 14 (6), 1897–1904. 10.1016/j.dsx.2020.09.029 PMC7521380 33007661

[B30] Waddell T. Bagur A. Cunha D. Thomaides‐Brears H. Banerjee R. Cuthbertson D. J. (2022). Greater ectopic fat deposition and liver fibroinflammation, and lower skeletal muscle mass in people with type 2 diabetes. Obesity 30, 1231–1238. 10.1002/oby.23425 35475573 PMC9321120

[B31] Xie J. Liu Y. Zeng X. Zhang W. Mei Z. (2017). A bayesian network model for predicting type 2 diabetes risk based on electronic health records. Mod. Phys. Lett. B 31 (19-21), 1740055. 10.1142/s0217984917400553

[B32] Zaffina C. Wyttenbach R. Pagnamenta A. Grasso R. F. Biroli M. Del Grande F. (2022). Body composition assessment: Comparison of quantitative values between magnetic resonance imaging and computed tomography. Quantitative imaging Med. Surg. 12 (2), 1450–1466. 10.21037/qims-21-619 PMC8739087 35111638

