# Supplementary material for: Bayesian networks and imaging-derived phenotypes highlight the role of fat deposition in COVID-19 hospitalisation risk
Source: Front Bioinform. 2023 May 24;3:1163430. doi: 10.3389/fbinf.2023.1163430 (PMC10244647; doi:10.3389/fbinf.2023.1163430)
Supplement: Supplementary file 1 [file DataSheet1.PDF]

Supplementary material

1. ***Overview of Bayesian-network methodology***
2. ***Overview of MR techniques and image analysis procedures***
3. ***Cohort flow diagram***
4. ***Discretisation thresholds***
5. ***Prediction accuracy***

1. *Overview of Bayesian networks*

Bayesian-networks (BN) are composed of 1) a directed acyclic graph (DAG) specifying conditional dependencies between network variables, and 2) a set of conditional probability distributions attached to each variable within the DAG. Formally, a DAG is expressed as G = (V, E), where V = {X_1, X_2, …, X_n} denotes the random variables of interest (in the present case, participant biomarkers such as obesity status), and where E is a set of directed edges relating pairs of variables in V. The directionality of an edge from X_i to X_j captures the flow of *information* between those two variables, where in that case the value of X_j is conditionally dependent on the value of X_i. Concretely, the structure of a DAG is comprised of three types of connections between variables: chains, forks, and colliders. Together these allow the reader to conveniently (and visually) detect interdependencies within the data.

In the example of Figure 1a, the directed edge from X to Z makes explicit that the value of Z is **conditionally dependent** on the value of X. Importantly, such graphical representation also provides a compact visualisation of **conditional independence** between variables, allowing direct cause and effect relationships to be explored.

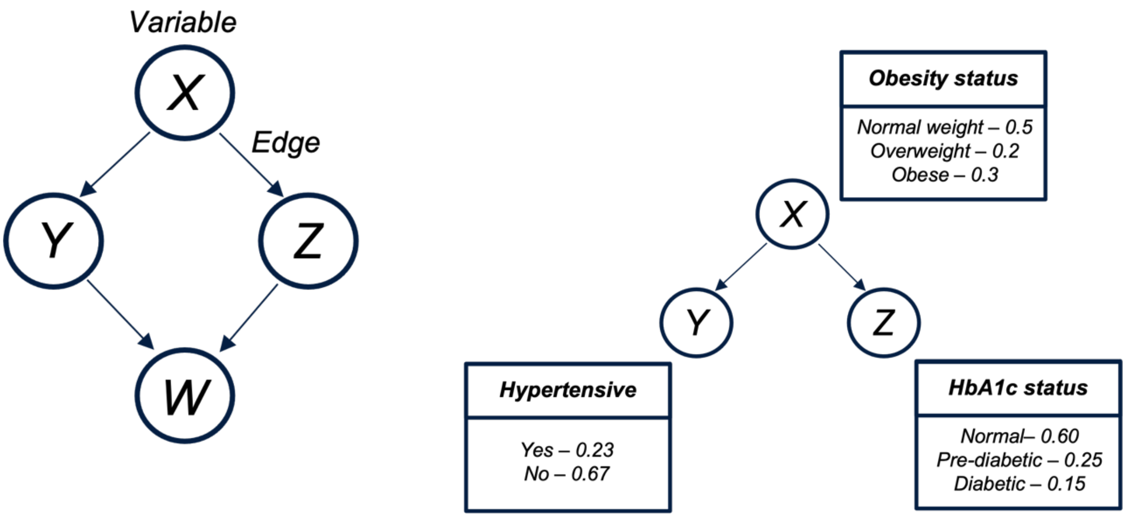

Figure 1a. (left) Example Bayesian-network where X, Z, Y and W denote 'variables' and the 'edges' denote the direction of causality; **Figure 1b**. (right) Example Bayesian-network with corresponding conditional probability tables.

<u>Bayesian-network construction</u>

Though a potentially powerful tool, special care needs to be taken when applying BNs to study *causality* in healthcare, for the presence and orientation of edges need to be consistent with medical knowledge. In the present study, the network structure was constructed in collaboration with medical experts, as follows. The score-based Hill-Climbing structure learning algorithm (1) with Bayesian Information Criterion (BIC) provided the initial, automated network construction, see Figure 2. This network structure was then adjusted by removing or reversing nonsensical edges, such as the edge from 'VAT to 'Age', and inserting edges based on domain knowledge gleaned from medical literature, such as the edge from 'Liver fat' to 'Hospitalisation'. Crucially, the incorporation of clinical knowledge in this network structure enables the modelling of *causal* relationships between variables, for the presence and direction of edges are not simply bias dependencies within the dataset. Such approach yields what is referred to as a 'semantic network'. Variable parameters were then fitted using the 'Bayes' method with uniform priors within the bn.fit function.

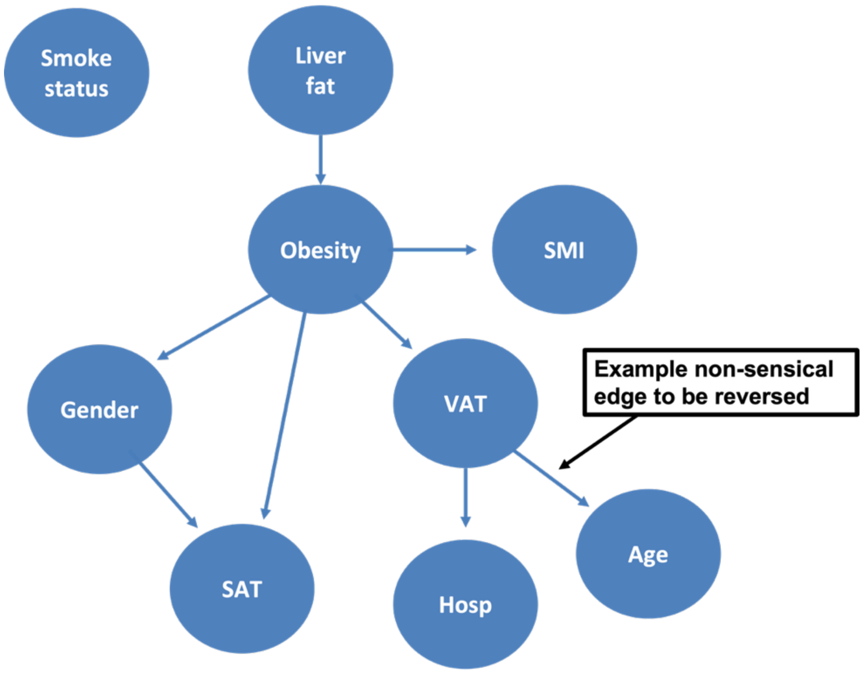

Figure 2. Bayesian-network structure derived from automated technique before manual adjustment. Here, we have highlighted an example non-sensical edge for reversal (for one's measure of VAT cannot affect their age).

Probabilistic inference

Concretely, a variable - x_1- is conditionally independent of its non-descendants *given* its parents within the network, denoted $P(x_1|\ Pa\ (x_1))$, with the value of x_1 conditionally dependent on that of its parent (Pa) node(s). Such conditional dependencies are factorised to form a joint probability distribution across the entire network via the chain rule:

$$P\ (x_1, x_2, \dots, x_n) = P\ (x_1)\ P\ (x_2|x_1) \dots P\ (x_n|x_1 x_2 \dots\ x_n{-}_1)$$

Such factorisation also allows a compact representation of the joint probability distribution of a specific variable, for x_1 and x_2 will not appear in each other's conditioning set if deemed conditionally independent given G.

Each variable within the Bayesian-network has a corresponding conditional probability table (CPT), which is learned from the data we fit to our network. These CPTs illustrate the conditional probability of each value state of that variable. For example, in Figure 1b. the

value states of ‘Obesity status’ are normal weight (0.5 = 50%), overweight (0.2 = 20%) and obese (0.3 = 30%), where the sum of each probability contained in the CPT must equal 1 (100%).

Conditional dependence (or causality) encoded within Bayesian-networks is especially critical in the context of biological systems, for each variable is considered conditionally independent of its non-descends given its parents. This allows the user to apply probabilistic inference and pose counterfactual ‘what if’ questions such as ‘what would happen to the probability of value *i* in variable Z (Z^i), given that I change the probability of value *X*?’. In the example of Figure 1b, such question could take the form of: ‘what is the change in the probability of diabetic HbA1c status, given that the probability of obesity increases to 50%, or decreases to 10%?’. We pose such questions by *intervening* on the network, where the user fixes the value of a specific variable(s), denoted as the ‘evidence’, and estimates the probability of an event, *given* the evidence.

We estimate such probabilities by performing conditional probability queries using the ‘cpquery’ function in bnlearn. Here, the probabilities were estimated using the likelihood weighting algorithm, a Monte Carlo approximation technique that uses importance sampling from the ‘mutilated network’ to estimate an event, given the fixed evidence. In the present study, the ‘event’ is the probability of hospitalisation following acute COVID-19, and the ‘evidence’ was the fixed values of the variables of interest, such as liver fat or obesity. This algorithm was selected given the relatively low sample size and confirmed hospitalisations. For more information on the application of ‘bnlearn’ for performing Bayesian-network analysis with examples, see (2).

2. *Overview of MR techniques and image analysis*

As part of the COVERSCAN study, all participants underwent an abdominal MRI assessment at two UK research imaging sites (Perspectum Ltd, Oxford; and Mayo Clinic Healthcare, London). Each scan lasted ~35 minutes where multi-organ data (liver, body composition) were collected using Siemens Healthcare MAGNETOM Aera 1.5T and MAGNETOM Vida 3T scanners at the Oxford and London locations, respectively.

Liver assessment

Liver MRI scans were analysed using Perspectum's LiverMultiScan technology. This software automatically delineates the liver from cT1 (corrected T1), T2* and proton density fat fraction (PDFF) (a measure of tissue fat content) image maps, excluding major vessels within the image section using a previously published deep-learning approach (3). Liver proton density fat fraction is measured as a percentage. For a full overview of MR and image analysis techniques see (4).

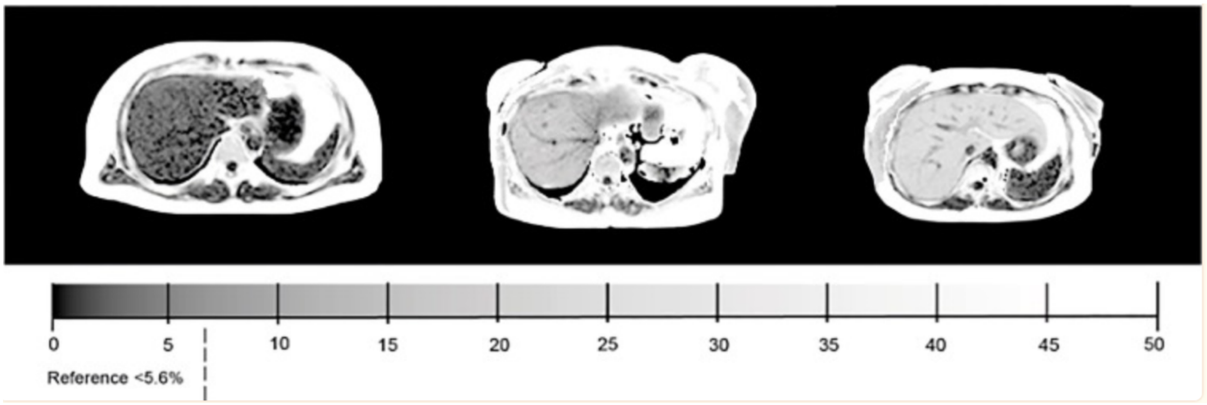

Figure 3. Example liver proton density fat fraction map.

Body composition

Subcutaneous adipose tissue (SAT), visceral adipose tissue (VAT) and skeletal muscle were delineated from a 2D MR slice positioned at the third lumbar (L3). This region was selected as it has shown to be strongly associated with whole-body skeletal muscle distribution and accurately estimate total SAT and VAT (5-7). VAT, SAT, and skeletal muscle were manually segmented from this 2D-slice by trained analysts using ITK-SNAP software and are measured in cm^2. See Figure 2 for an example body composition segmentation.

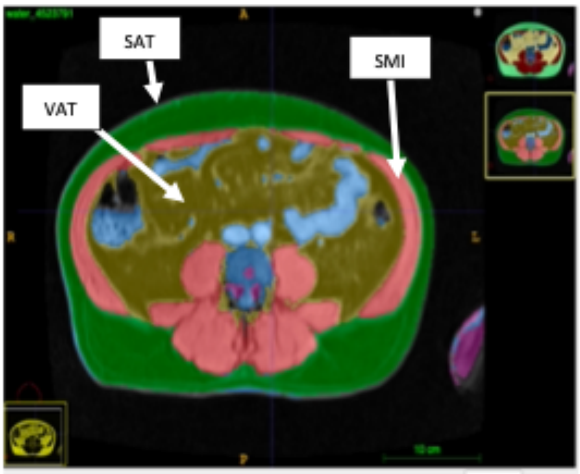

Figure 4. Example 2D MR slice positioned at the third-lumbar vertebrae. VAT, SAT, and SMI show example manual segmentations.

3. *Cohort flow diagram from COVERSCAN study*

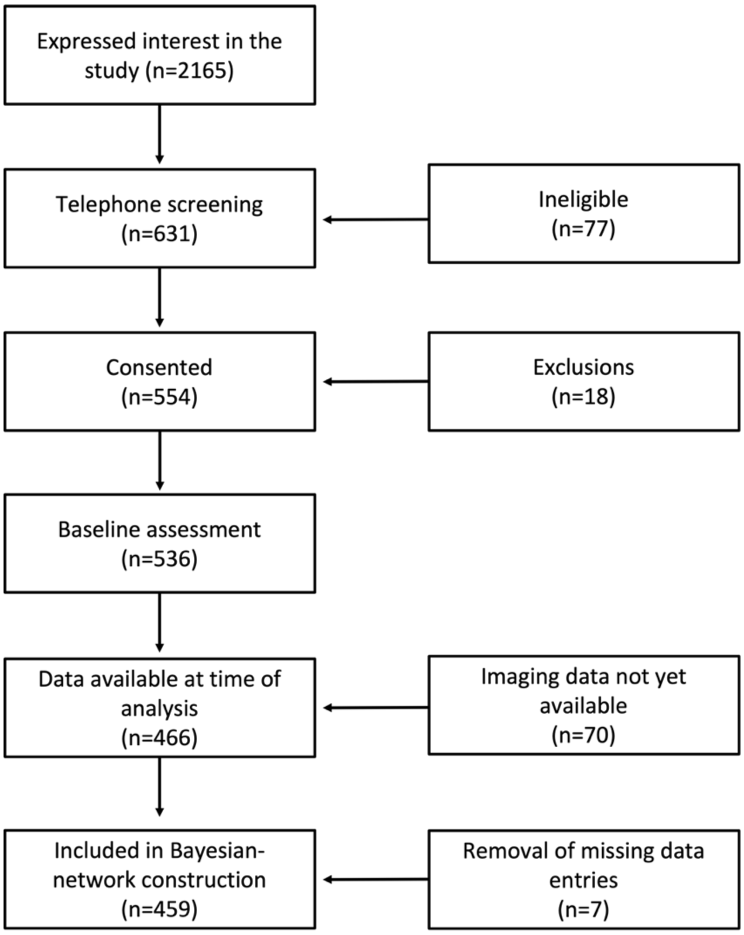

Figure 5. Cohort flow diagram.

4. Discretisation thresholds

Supplementary Table 1. Discretisation thresholds used for construction of Bayesian-network and probabilistic inference.

Variable	Discretisation
Liver PDFF (%)<br><br>Relevant publication:<br><br>Roca-Fernández, A., Dennis, A., Nicholls, R., McGonigle, J., Kelly, M., Banerjee, R., Banerjee, A. and Sanyal, A.J., 2021. Hepatic steatosis, rather than underlying obesity, increases the risk of infection and hospitalization for COVID-19. *Frontiers in Medicine*, *8*, p.636637.	<5% - normal (n = 370)<br>5-10% - mild steatosis (n = 40)<br>>10% - severe steatosis (n = 49)
VAT (cm^2)<br><br>Elevated measures defined as values greater than the 75th percentile derived from descriptive statistics.	**Male**<br>Normal - <210(cm^2)<br>Elevated - >210(cm^2)<br><br>**Female**<br>Normal - <140(cm^2)<br>Elevated - >140(cm^2)<br><br>**Total**<br>Normal (n = 364)<br>Elevated (n = 95)
SAT (cm^2)<br><br>Elevated measures defined as values greater than the 75th percentile derived from descriptive statistics.	**Male**<br>Normal - <220(cm^2)<br>Elevated - >220(cm^2)<br><br>**Female**<br>Normal - <400(cm^2)<br>Elevated - >400(cm^2)<br><br>**Total**<br>Normal (n = 312)<br>Elevated (n = 147)
SMI (cm^2/m)<br><br>Reduced measures defined as values less than the 25th percentile derived from descriptive statistics.	**Male**<br>Normal- >44(cm^2/m)<br>Reduced - <44(cm^2/m)<br><br>**Female**<br>Normal - >31(cm^2/m)<br>Reduced - <31(cm^2/m)<br><br>**Total**<br>Normal (n = 422)

	Reduced (n = 37)
Body mass index (kg/m^2)	Normal weight - <25(kg/m^2) (n = 210)<br>Overweight – 25-30 (kg/m^2) (n = 148)<br>Obese – >30(kg/m^2) (n = 101)
Age (yrs)	<40yrs (n = 146)<br>40-50yrs (n = 163)<br>50-60yrs (n = 113)<br>>60yrs (n = 37)

5. ***Additional predictive performance of classifying hospitalisation status.***

Supplementary Table 2. Additional predictive performance measures (precision, recall, Matthews Correlation Coefficient) of classification algorithms.

	**Precision**	**Recall**	**MCC**
**Bayesian-network**	0.98	0.88	0.35
**Naïve Bayes**	0.92	0.89	0.26
**Logistic regression**	0.85	0.85	-0.005
**Decision tree**	0.81	0.88	0.13

References

1. Selman, B. and Gomes, C.P., 2006. Hill-climbing search. Encyclopedia of cognitive science, 81, p.82.
2. Scutari M, Denis J (2021). *Bayesian Networks with Examples in R*, 2nd edition. Chapman and Hall, Boca Raton. ISBN 978-0367366513.
3. Irving B, Hutton C, Dennis A, et al. Deep quantitative liver segmentation and vessel exclusion to assist in liver assessment. In: Medical Image Understanding and Analysis: 21st Conference, MIUA 2017, Edinburgh, UK, July 11-13, 2017, Proceedings. Springer International Publishing; 2017: 663-673.
4. Waddell, T., Bagur, A., Cunha, D., Thomaides-Brears, H., Banerjee, R., Cuthbertson, D.J., Brown, E., Cusi, K., Després, J.P. and Brady, M., 2022. Greater ectopic fat deposition and liver fibroinflammation and lower skeletal muscle mass in people with type 2 diabetes. *Obesity*, *30*(6), pp.1231-1238.

5. Demerath, E.W., Shen, W., Lee, M., Choh, A.C., Czerwinski, S.A., Siervogel, R.M. and Towne, B., 2007. Approximation of total visceral adipose tissue with a single magnetic resonance image. *The American journal of clinical nutrition*, *85*(2), pp.362-368.
6. Shen, W., Punyanitya, M., Wang, Z., Gallagher, D., St.-Onge, M.P., Albu, J., Heymsfield, S.B. and Heshka, S., 2004. Total body skeletal muscle and adipose tissue volumes: estimation from a single abdominal cross-sectional image. *Journal of applied physiology*, *97*(6), pp.2333-2338.
7. Schweitzer, L., Geisler, C., Pourhassan, M., Braun, W., Glüer, C.C., Bosy-Westphal, A. and Müller, M.J., 2015. What is the best reference site for a single MRI slice to assess whole-body skeletal muscle and adipose tissue volumes in healthy adults?. *The American journal of clinical nutrition*, *102*(1), pp.58-65.
